# The lattice dislocation trapping mechanism at the ferrite/cementite interface in the Isaichev orientation relationship

**DOI:** 10.1038/s41598-021-88544-6

**Published:** 2021-04-29

**Authors:** Jaemin Kim, Hadi Ghaffarian, Keonwook Kang

**Affiliations:** 1 Department of Mechanical Engineering & KI for the NanoCentury, Korea Advanced Institute of Science and Technology, Daejeon, 34141 Republic of Korea; 2 Department of Mechanical Engineering, Yonsei University, Seoul, 03722 Republic of Korea

**Keywords:** Materials science, Structural materials, Theory and computation

## Abstract

We analyze the lattice dislocation trapping mechanism at the ferrite/cementite interface of the Isaichev orientation relationship by atomistic simulations combined with the anisotropic linear elasticity theory and disregistry analysis. We find that the lattice dislocation trapping ability is varied by initial position of the lattice dislocation. The lattice dislocation near the interface is attracted to the interface by the image force generated by the interface shear, while the lattice dislocation located far is either attracted to or repelled from the interface, or even oscillates around the introduced position, depending on the combination of the stress field induced by the misfit dislocation array and the image stress field induced by the lattice dislocation.

Pearlitic steel has been utilized for many engineering and industrial applications for its excellent mechanical properties. It is formed by the diffusive eutectoid reaction, a decomposition process of a face-centered cubic (FCC) austenite containing small amounts of carbon atoms into a body-centered cubic (BCC) ferrite and orthorhombic cementite (Fe_3_C). It consists of three-dimensionally interwoven ferrite and cementite phases between which crystallographic orientation relationships (ORs)^1–10^ of IS (Isaichev), Near BA (Near Bagaryatsky) and Near PP (Near Pitsch–Petch) are dominantly observed according to recent experimental and computational studies^4,8^.

Numerous studies^11–14^ have been performed to enhance the mechanical properties of the pearlitic steel by reducing the interlamellar spacing of the lamellar structure. It is reported that pearlitic steel with a fine lamellar structure of a few tens to hundreds of nanometers can be formed by controlling the experimental conditions during the diffusive eutectoid reaction^11,14^. At such a small scale where the interface-to-volume fraction increases significantly, the overall mechanical properties of pearlitic steel are greatly influenced by the ferrite-cementite interface (FCI)^12,15–22^. Since the characteristics of the FCI change with its OR^4^, the interaction between the interface and defects can be very different for each OR. Moreover, many efforts^23–26^ have been made to form a pearlitic steel with specific crystallographic ORs by changing the experimental conditions, which concluded that the occurrence of specific OR can be controlled by carbon contents of the system^23^, heat treatment condition^24^ and applying a magnetic field during phase transformation^25,26^. This implies that the overall mechanical properties of nanostructured pearlitic steel are tunable with the adjustment of processing conditions for obtaining specific ORs.

Several researchers^27,28^ reported that the strength of the multi-layered metallic composite with nanoscale layer thickness is determined by the lattice dislocation trapping mechanism at the interface. They claimed that the stress field due to the lattice dislocation near the interface causes the shearing of a weak interface, and the lattice dislocation is absorbed to the interface by the image force from the interfacial shear. Similarly, in pearlitic steel, a few studies reported that the FCI acts as a strong sink to dislocation when the interlamellar spacing is smaller than critical thickness^16,21^. The samples had high dislocation density near the FCI but few dislocations exist in the ferrite layer. However, no prior study has been carried out to understand the lattice dislocation trapping mechanism based on the interface-dislocation interaction in pearlitic steel.

Here, we performed a combined theoretical and atomistic simulation study to analyze the lattice dislocation trapping mechanism at the FCI for the IS OR. We constructed a ferrite/cementite bilayer model and observed the motion of the lattice dislocation embedded initially at various positions within the ferrite layer during the 300 K molecular dynamics (MD) simulations. We used disregistry analysis to characterize the interface shear and utilized anisotropic linear elasticity theory to compute the spatial distribution of the stress field. We concluded that the motion of the lattice dislocation located far away from the interface is governed by the combination of the stress field induced by misfit dislocations and the image stress field induced by the lattice dislocation due to the presence of the interface, while the motion of the lattice dislocation located near the interface is governed solely by the image force induced by the interface shear.

We chose the IS OR because it has the weakest in-plane shear strength among IS, Near BA and Near PP ORs^29^, and hence the lattice dislocation trapping mechanism can be easily induced by the interface shear. In this study, we put the *x*_2_-axis paralleled to the interface normal and imposed periodic boundary conditions in *x*_1_ and *x*_3_ directions, as shown in Fig. 1a. To satisfy IS OR, we set the crystallographic orientation as **x**_1_ = [010]_c_||[$\bar{1}{\text{11}}$]_f_, **x**_2_ = $(\bar{1}{\text{01}})$_c_||$(\bar{1}\bar{2}{\text{1}})$_f_ and **x**_3_ = **x**_1_ $\times$ **x**_2_ for perfect ferrite and cementite blocks. The subscripts f and c represent the ferrite and cementite phases, respectively. The modified embedded-atom method (MEAM) potential^30^ is employed to consider the metallic bond between Fe atoms and covalent bond between Fe and C atoms. The lattice parameter of ferrite is *a*_f_ = 2.851 Å and the lattice parameters of cementite are *a*_c_ = 4.470 Å, *b*_c_ = 5.088 Å and *c*_c_ = 6.670 Å.

**Figure 1:**
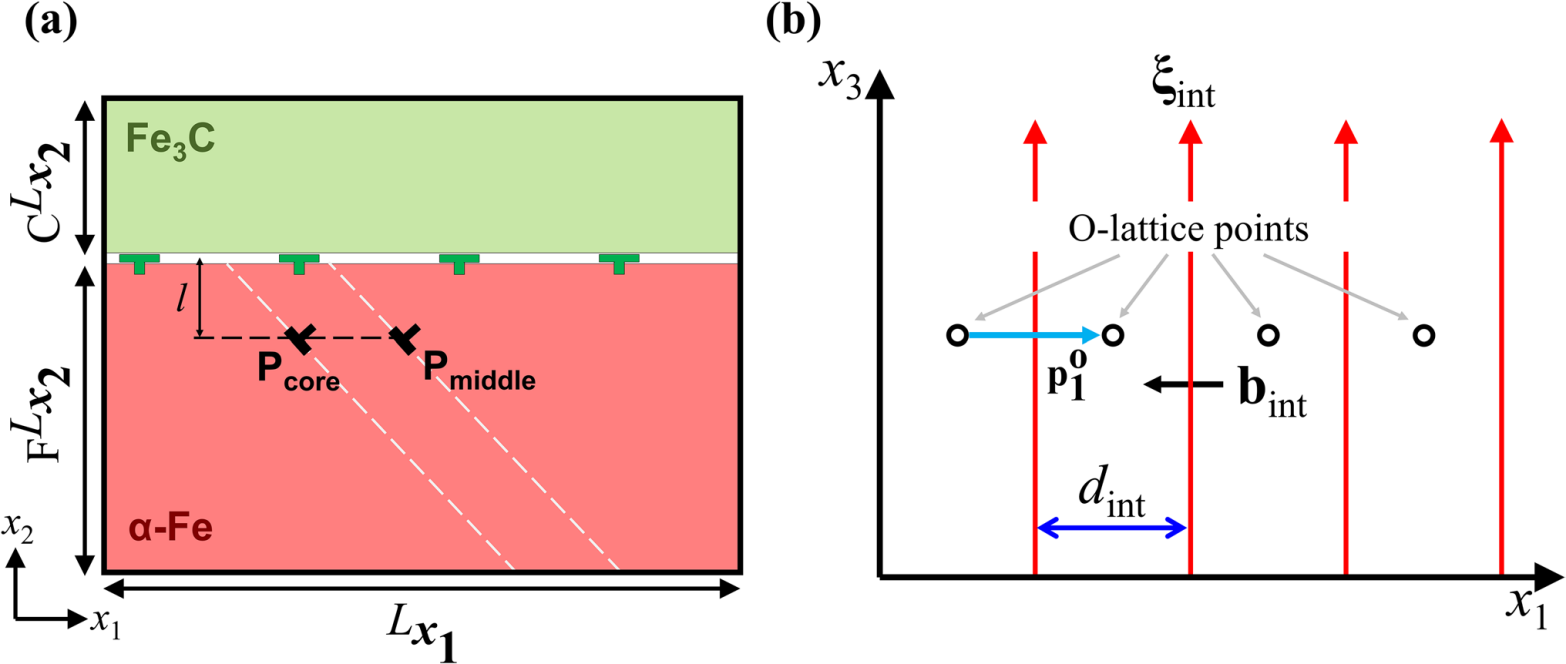
Schematic representation of (**a**) the ferrite/cementite bilayer model for an Isaichev (IS) orientation relationships with a straight lattice dislocation in the ferrite layer and (**b**) the arrays of misfit dislocations and its O-lattice vector.

The perfect ferrite and cementite blocks with the given orientation were generated to yield the misfit strain as small as possible with affordable computational cost. The misfit strains in *x*_1_ and *x*_3_ directions were ${\upvarepsilon }_{{{x}}_{1}}^{\text{misfit}}=$ 2.08 $\times$ 10^–6^ and ${\upvarepsilon }_{{{x}}_{3}}^{\text{misfit}}=$ 4.3 $\times$ 10^–3^, respectively. The biaxial strain was iteratively applied to the ferrite and cementite blocks separately, until the geometric compatibility and mechanical equilibrium are satisfied^31^. Then, the strained ferrite and cementite blocks were assembled into un-relaxed ferrite/cementite bilayer. In order to relax the FCI structure, we performed an energy minimization at 0 K using the conjugate gradient method, followed by the simulated annealing in which temperature is lowered from 800 to 10 K with geometric cooling manner for 100 ps using Nosé–Hoover isobaric–isothermal (NPT) ensemble. Another round of conjugate gradient energy minimization at 0 K is performed to obtain initial FCI structure. The final dimension of the ferrite/cementite bilayer for IS OR is ${{L}}_{{{x}}_{1}}={335.67}$ Å, ${}_{\text{F}}{{{L}}}_{{{x}}_{2}}={166.7}$ Å, ${}_{\text{C}}{{{L}}}_{{{x}}_{2}}={80.46}$ Å and ${{L}}_{{{x}}_{3}}={32.1}{9}$ Å as shown in Fig. 1a. The Burgers vector, line orientation and line spacing of the arrays of misfit dislocations were **b**_int_ = [− 2.51, 0.00, 0.00] Å, **ξ**_int_ = [0.00 0.00 1.00] and *d*_int_ = 83.92 Å, respectively (see Fig. 1b).

Once the ferrite/cementite bilayer of IS OR is constructed, a straight mixed lattice dislocation is introduced at various positions within the ferrite layer using the displacement field solution derived from the linear elasticity theory^32^ (see Fig. 1a). The **P**_middle_ represents a position of vertical distance *l* from the middle of the two misfit dislocations at the interface, while the **P**_core_ represents a position of vertical distance *l* from the misfit dislocation core at the interface. The Burgers vector and line orientation of the lattice dislocation are **b**_lat_ = [0.82 − 1.16 2.01] Å and **ξ**_lat_ = [0.00 0.00 1.00], respectively, and these correspond to a straight mixed dislocation with 1/2[111]$\left(\overline{1 }01\right)$ Burgers vector. In order to observe the spontaneous motion of the lattice dislocation, we carried out the MD simulations at 300 K using NPT ensemble to eliminate all the average stress components. We considered 18 $\times$ 11 grid points in the ferrite layer as a target box, and ran multiple simulations after introducing a straight lattice dislocation at each grid point. The grid spacing, which is much smaller than the spacing of the misfit dislocations (*d*_int_ = 83.75 Å), is 4.93 Å and 6.98 Å in *x*_1_ and *x*_2_ directions, respectively. The lattice dislocation either approaches toward the interface, runs away from the interface, or is stationary depending on the initial position.

In order to analyze the motion of the lattice dislocation within the ferrite layer, we computed the Peach–Kohler (P–K) force on the lattice dislocation. To compute the P–K force, it is necessary to compute both the stress field induced by the misfit dislocations, ${}_{{\upkappa }}^{{}} {\varvec{\upsigma}}_{{{\text{int}}}} \left( {x_{1} , x_{2} } \right)$, and the image stress field induced by the lattice dislocation due to the presence of the interface^33^, ${}_{{\upkappa }}^{{}} {\varvec{\upsigma}}_{{{\text{img}}}} \left( {x_{1} , x_{2} } \right)$. The subscript κ represents either ferrite (F) or cementite (C) phase. Although the stress intensity is varied by the dislocation core-width^34^, we assumed the dislocations as the Volterra dislocation to solve the anisotropic linear elasticity theory^35–37^ because the dislocation core-width does not significantly change the overall spatial stress distribution except for core-region (2*w* = 26 Å, *w* represents half-width of misfit dislocation core). For computing the stress field induced by the misfit dislocations ${}_{{\upkappa }}^{{}} {\varvec{\upsigma}}_{{{\text{int}}}} \left( {x_{1} , x_{2} } \right)$, we assumed that (i) the interface atoms experience stepwise relative displacement ${\Delta }{\mathbf{u}}_{{{\text{int}}}}$ near the misfit dislocations (see Fig. 2a), (ii) no net traction exists along the interface, and (iii) the far-field stress induced by the arrays of Volterra dislocations and coherency stress satisfy the mechanical equilibrium^37^. The stepwise relative displacement ${\Delta }{\mathbf{u}}_{{{\text{int}}}}$ is expressed as a combination of the affine ${\Delta }{\mathbf{u}}_{{{\text{aff}}}} = {}_{{\text{C}}}^{{}} {\mathbf{u}}_{{{\text{aff}}}} - {}_{{\text{F}}}^{{}} {\mathbf{u}}_{{{\text{aff}}}}$ and non-affine ${\Delta }{\mathbf{u}}_{{\text{non-aff}}} = {}_{{\text{C}}}^{{}} {\mathbf{u}}_{{\text{non-aff}}} - {}_{{\text{F}}}^{{}} {\mathbf{u}}_{{\text{non-aff}}}$ relative displacement, where ${}_{{\upkappa }}^{{}} {\mathbf{u}}_{{{\text{aff}}}}$ and ${}_{{\upkappa }}^{{}} {\mathbf{u}}_{{\text{non-aff}}}$ represent the affine and non-affine displacements in κ-phase, respectively. The affine relative displacement $\Delta {\mathbf{u}}_{{{\text{aff}}}}$ is determined from the coherency strain to satisfy macroscopic geometric compatibility between ferrite and cementite blocks. The non-affine relative displacement $\Delta {\mathbf{u}}_{{\text{non-aff}}}$ is given by the non-uniform relative displacement to minimize the atomic mismatch between ferrite and cementite atomic layers. For computing the stress field induced by the lattice dislocation ${}_{{\upkappa }}^{{}} {\varvec{\upsigma}}_{{{\text{lat}}}} \left( {x_{1} , x_{2} } \right)$, we assumed the displacement continuity across the interface and the zero net traction along the interface^35^. The spatial stress distribution is depicted in Fig. 2b–d. The detailed descriptions to compute the stress field solution can be found in the Supplementary Material. The stress field solution ${}_{{\upkappa }}^{{}} {\varvec{\upsigma}}_{{{\text{lat}}}} \left( {x_{1} , x_{2} } \right)$ induced by the lattice dislocation can be expressed as ${}_{{\upkappa }}^{{}} {\varvec{\upsigma}}_{{{\text{lat}}}} \left( {x_{1} , x_{2} } \right) = {}_{{\upkappa }}^{{}} {\varvec{\upsigma}}_{{{\text{inf}}}} \left( {x_{1} , x_{2} } \right) + {}_{{\upkappa }}^{{}} {\varvec{\upsigma}}_{{{\text{img}}}} \left( {x_{1} , x_{2} } \right)$, where ${}_{{\upkappa }}^{{}} {\varvec{\upsigma}}_{{{\text{inf}}}} \left( {x_{1} , x_{2} } \right)$ represents the infinite stress field solution when the lattice dislocation is located in the infinite crystal, and ${}_{{\upkappa }}^{{}} {\varvec{\upsigma}}_{{{\text{img}}}} \left( {x_{1} , x_{2} } \right)$ represents the image stress field solution, which is generated to satisfy the boundary conditions at the interface. When the P–K force on the lattice dislocation is computed, the infinite stress field solution ${}_{{\text{F}}}^{{}} {\varvec{\upsigma}}_{{{\text{inf}}}}$ was neglected because it does not affect the motion of the lattice dislocation itself^35^. In addition, the ferrite layer experiences external strains along *x*- and *z*-directions due to different thermal expansion coefficient of ferrite and cementite phases at 300 K, resulting in developing a non-zero stress state (${}_{{\text{F}}}^{{}} {\varvec{\upsigma}}_{{{\text{ext}}}}$) in the ferrite layer, which must be taken into account for calculating P–K force. Our analysis shows that the ferrite layer's external strain along *x*- and *z*-directions is equal to 7.07 × 10^–4^ and − 1.97 × 10^–4^, respectively. Therefore, we computed the P–K force on the lattice dislocation within the ferrite layer ${}_{{\text{F}}}^{{}} {\mathbf{f}}_{{{\text{lat}}}}^{{{\text{PK}}}}$ by

1 $$\begin{aligned} {}_{{\text{F}}}^{{}} {\mathbf{f}}_{{{\text{lat}}}}^{{{\text{PK}}}} &= \left\{ {\left( {{}_{{\text{F}}}^{{}} {{\varvec{\upsigma}}}_{{{\text{int}}}} + {}_{{\text{F}}}^{{}} {{\varvec{\upsigma}}}_{{{\text{img}}}} + {}_{{\text{F}}}^{{}} {{\varvec{\upsigma}}}_{{{\text{ext}}}} } \right){ } \cdot { }{\mathbf{b}}_{{{\text{lat}}}} } \right\} \times {\upxi }_{{{\text{lat}}}}\\ {}_{{\text{F}}}^{{}} f_{{\text{d}}}^{{{\text{PK}}}}& = {\mathbf{d}}{ } \cdot { }{}_{{\text{F}}}^{{}} {\mathbf{f}}_{{{\text{lat}}}}^{{{\text{PK}}}}\end{aligned} $$

where ${}_{{\text{F}}}^{{}} f_{{\text{d}}}^{{{\text{PK}}}}$ is the resolved P–K force on the lattice dislocation within the ferrite layer into the direction of unit vector **d**. ${\mathbf{d}}$ = [− 0.58, 0.82, 0.00] is a direction vector in the $\left(\overline{1 }01\right)$ slip plane as shown in Fig. 3a. When ${}_{{\text{F}}}^{{}} f_{{\text{d}}}^{{{\text{PK}}}}$ has positive/negative value, then the lattice dislocation is subject to the interface's attractive/repulsive force. It is noticed that the external stress in ferrite layer ${}_{{\text{F}}}^{{}} {\varvec{\upsigma}}_{{{\text{ext}}}}$ generates − 0.0073 N/m repulsive force on the lattice dislocation which only contributes 5% of the average P–K force in the target box but it should be included for correct prediction of the borderline between the attractive and repulsive regions.

**Figure 2:**
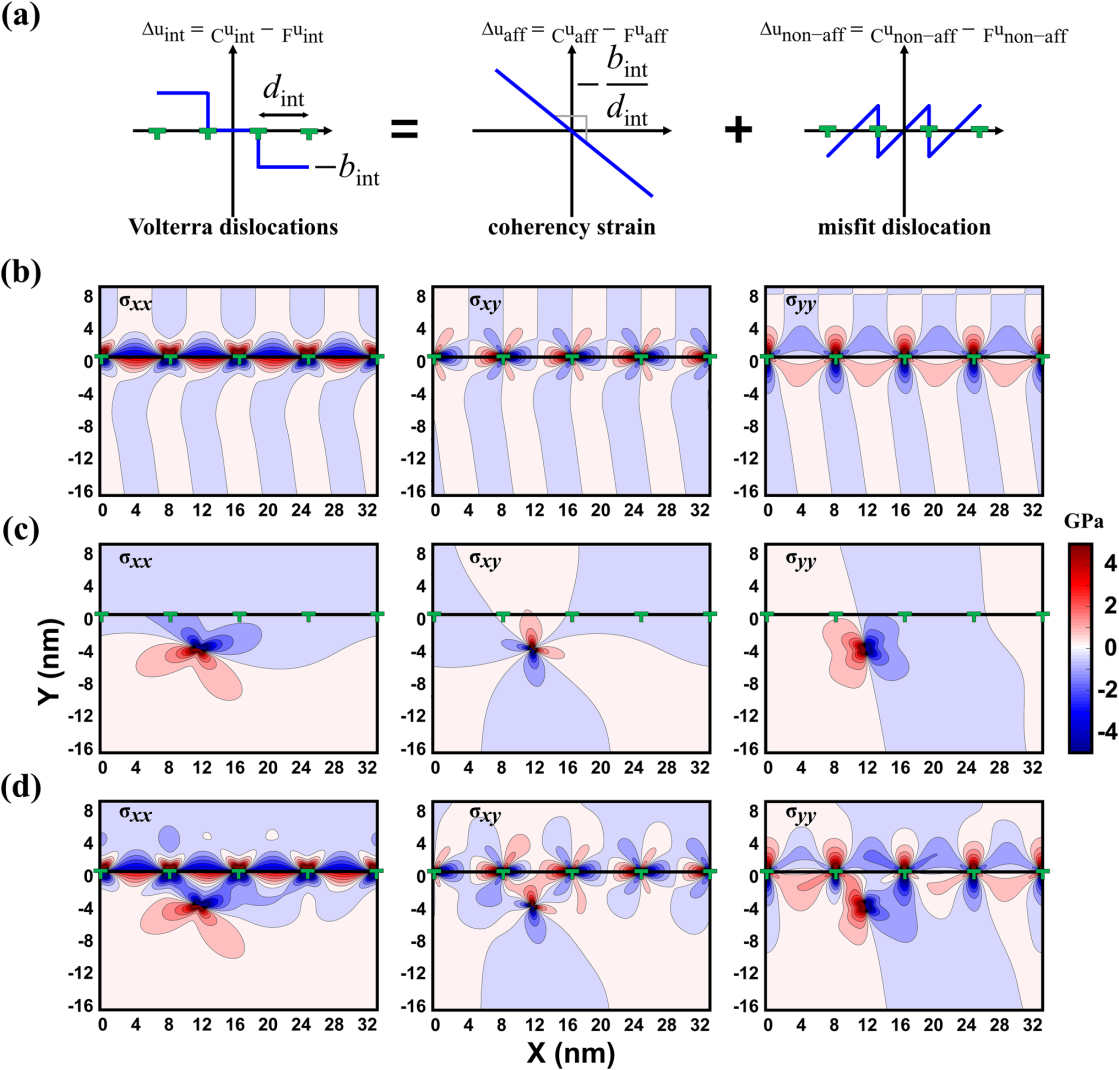
(**a**) The relative displacement field of arrays of Volterra dislocations at the interface, (**b**) the spatial stress distribution induced by the arrays of misfit dislocations at the interface, (**c**) the spatial stress distribution induced by the lattice dislocation within the ferrite layer and (**d**) the spatial stress distribution induced by the arrays of misfit dislocations and lattice dislocation.

**Figure 3:**
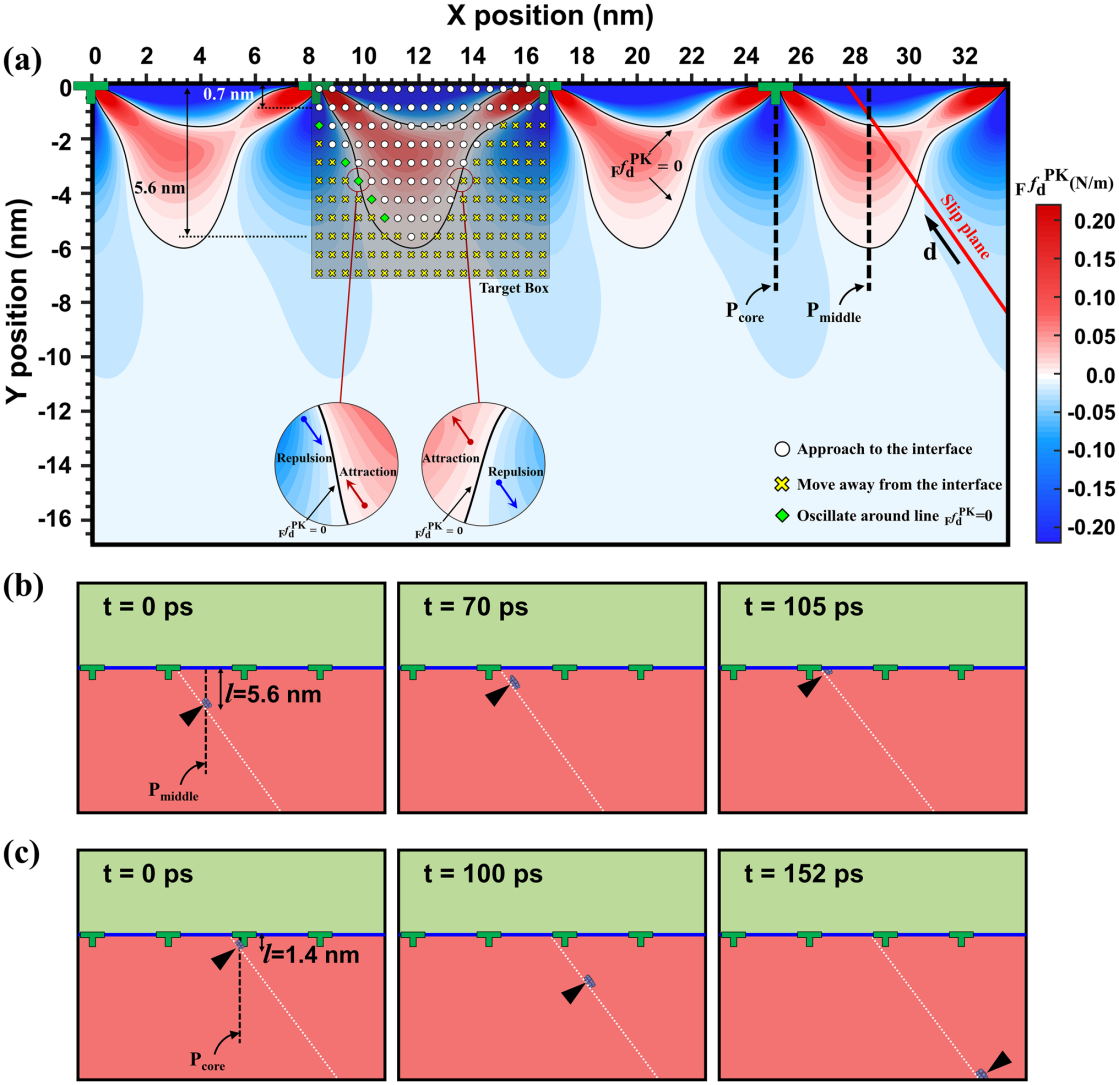
(**a**) Contour plot of resolved P–K force within the ferrite layer. The target box represents MD simulation results of lattice dislocation trapping ability with respect to the initial position of lattice dislocation. Solid black and red lines represent ${}_{{\text{F}}}^{{}} f_{{\text{d}}}^{{{\text{PK}}}} = 0$ borderline and the slip plane, respectively. White circle, yellow x-mark, and green square in the target box indicate that the lattice dislocation is attracting by the interface, moving away from the interface and oscillating around the borderline, respectively. The insets reveal the direction of P–K force near the borderline at the left and right sides of the target box, providing metastable and unstable trapping sites, respectively. (**b**) Trajectory of lattice dislocation trapping mechanism for dislocation located at P_middle_ region with *l* = 5.6 nm. (**c**) Trajectory of lattice dislocation repulsion mechanism for dislocation located at P_core_ region with *l* = 1.4 nm. Solid blue and dashed white lines in (**b**) and (**c**) represent the ferrite/cementite interface and the slip plane in the ferrite layer, respectively. The black triangles in (**b**) and (**c**) indicate the position of lattice dislocation.

We then compare the lattice dislocation motion observed in MD simulations with the theoretical prediction, as shown in Fig. 3a. First, when the vertical distance *l* is beyond 5.6 nm, the lattice dislocation always escapes toward the bottom surface regardless of initial position in MD simulations due to the repulsive P–K force. The image stress from the free surface was not accounted in our P–K force calculation and it actually is another source of repulsion. Nonetheless, the prediction power of our model is still impressive and we think that the simulation cell is large enough to neglect the image stress contribution.

Second, when the lattice dislocation is located at the intermediate distance (0.7 < *l* ≤ 5.6 nm), the lattice dislocation moves toward or away from the interface, depending on its initial position. For instance, the lattice dislocation initially located at **P**_middle_ position moves to the interface as shown in Fig. 3b, while the lattice dislocation at the **P**_core_ position runs away (see Fig. 3c). Simulation results imply that the dislocation trapping ability depends on the initial position of the lattice dislocation. The lattice dislocation at the **P**_middle_ position is more prone to be attracted by the interface than the lattice dislocation at the **P**_core_ position. The overall contour of the resolved P–K force matches well with the MD simulations within the intermediate range. We note that the effect of lattice resistance was not significant for the mixed dislocation due to thermal fluctuation. The upper limit of the Peierls stress, i.e. the lattice resistance of pure screw dislocation at 300 K, is predicted to be 400 MPa with the present MEAM potential. Our analysis also shows that the trapping ability of FCI is mainly controlled with the edge component of lattice dislocation rather than the screw component (see supplementary materials for more details). The present elastic field solution can also predict dislocation trapping within the cementite layer. However, as a brittle phase, cementite has limited slip systems confined on (100) and (001) planes^38^ with relatively high Peierls stress for mixed dislocation due to increased lattice resistance. In such cases, dislocation glide motion is difficult, and hence the dislocation trapping can be limited from the cementite side.

In addition, the lattice dislocation at some initial positions is neither absorbed toward nor repels from the interface during MD simulation up to 600 ps, showing stable oscillation near the ${}_{{\text{F}}}^{{}} f_{{\text{d}}}^{{{\text{PK}}}} = 0$ borderline because of balancing between attractive and repulsive forces. Those stationary positions were only found at the left region of the target box (See green diamond markers in Fig. 3a). According to Eq. (1), it is noticeable that the contour plot of Fig. 3a is the contour plot showing the magnitude of P–K force along the direction vector **d** on the slip plane. Considering the orientation of P–K force, for the lattice dislocation located at the left side of the target box and near the ${}_{{\text{F}}}^{{}} f_{{\text{d}}}^{{{\text{PK}}}} = 0$ borderline, both attractive and repulsive forces push the lattice dislocation toward the ${}_{{\text{F}}}^{{}} f_{{\text{d}}}^{{{\text{PK}}}} = 0$ borderline, making it a metastable trapping site. In contrast, at the right side of target box, the net forces move the lattice dislocation away from the borderline, producing an unstable trapping site. On the other hand, all the observed stationary points are almost located on the same slip plane indicating the final trapping point on the ${}_{{\text{F}}}^{{}} f_{{\text{d}}}^{{{\text{PK}}}} = 0$ borderline is same for all of them (see Supplementary movies for more details).

Interestingly, for the initial location very close to the interface (*l* ≤ 0.7 nm), the lattice dislocation approaches to the interface regardless of the sign of the resolve P–K force. The previous studies^27,28^ suggest that the lattice dislocation trapping mechanism at the weak interface can be activated by the interface shear caused by the stress field from the lattice dislocation. Additional MD simulation and disregistry analysis were carried out to analyze the motion of the lattice dislocation for the **P**_middle_ and **P**_core_ positions for *l* = 0.7 nm based on the interface shear. To obtain the clear 2D disregistry, we performed MD simulations for the **P**_middle_ and **P**_core_ positions with a straight lattice dislocation at 50 K (see Fig. 4a,c). From the 2D disregistry maps as shown in Fig. 4b,d, it can be seen that the interface shear readily occurs even though the lattice dislocation does not reach the interface. This interface shear is originated by the shear stress component $\tau_{xy}$ induced by the lattice dislocation because the FCI for IS OR has extremely low interfacial shear strength in $\tau_{xy}$-direction (around one hundred megapascal)^29^. Once the interface shear occurs, the perfect interface assumption used to obtain an anisotropic elastic field solution does not hold, and the image force is generated to satisfy the stress boundary condition limited to interfacial shear strength of the FCI of IS OR. By this image force, the lattice dislocation near the interface is subject to attractive force to the interface. After the interface shear, the lattice dislocation is absorbed at the interface, and consequently, the core structure of the lattice dislocation spreads along the interface (see Fig. 4b,d). It implies that the lattice dislocation cannot escape the interface easily because the core structure of absorbed lattice dislocation spreads along the interface. The excellent lattice dislocation trapping ability of the FCI for IS OR may contribute to enhance the strength of the nanostructured pearlitic steel^27,28^.

**Figure 4:**
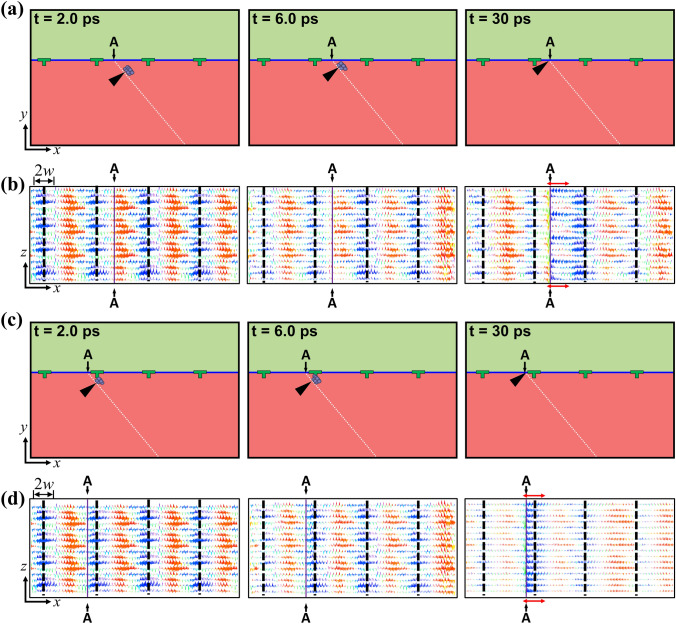
Trajectory of lattice dislocation trapping for the lattice dislocation located at (**a**) P_middle_ and (**c**) P_core_ positions as equilibration time increases at 50 K. Corresponded 2D disregistry map of interface atoms for the lattice dislocation located at (**b**) P_middle_ and (**d**) P_core_ positions. The vertical distance of lattice dislocation in (**a**) and (**c**) is 0.7 nm. The equilibration simulation is carried out at 50 K to compute the clear disregistry plot. The black triangle in (**a**) and (**c**) indicates the position of the lattice dislocation. Solid blue and dashed white lines in (**a**) and (**c**) indicate the ferrite/cementite interface and the slip plane in the ferrite layer, respectively. The black dashed line and purple solid line in (**b**) and (**d**) represent misfit dislocation line and slip plane trace, respectively. 2*w* and red arrow in (**b**) and (**d**) represent the core width of misfit dislocation and core spreading of lattice dislocation along the interface, respectively.

In summary, we investigated the lattice dislocation trapping ability of the FCI for IS OR based on the MD simulations combined with anisotropic elasticity theory and disregistry analysis. From the MD simulations, we find that the lattice dislocation trapping ability is varied by the initial position of the lattice dislocation. From the disregistry analysis and anisotropic elasticity theory, we conclude that the lattice dislocation near the interface approaches to the interface by the image force induced by the interface shear, while the lattice dislocation far away from the interface approaches/repels to the interface by both the stress field induced by the misfit dislocations and the image stress field induced by the lattice dislocation due to the presence of the interface.

## Supplementary information

Supplementary Information 1.

Supplementary Video 1.

Supplementary Video 2.

Supplementary Video 3.

Supplementary Video 4.

Supplementary Video 5.

## Data Availability

The data that support the findings of this study are available from the corresponding author upon reasonable request.
