# Supplementary material for: The lattice dislocation trapping mechanism at the ferrite/cementite interface in the Isaichev orientation relationship
Source: Sci Rep. 2021 Apr 29;11:9324. doi: 10.1038/s41598-021-88544-6 (PMC8085030; doi:10.1038/s41598-021-88544-6)
Supplement: Supplementary file 1 — Supplementary Information 1. [file 41598_2021_88544_MOESM1_ESM.docx]

Supplementary Information

**The lattice dislocation trapping mechanism at the ferrite/cementite interface in the Isaichev orientation relationship**

Jaemin Kim^a,†^, Hadi Ghaffarian^a,b,†^, Keonwook Kang^b,*^

^a^ Department of Mechanical Engineering & KI for the NanoCentury, Korea Advanced Institute of Science and Technology, Daejeon 34141, Republic of Korea

^b^ Department of Mechanical Engineering, Yonsei University, Seoul 03722, Republic of Korea

^†^ Jaemin Kim and Hadi Ghaffarian contributed equally to this work

^*^ Corresponding author. Tel.: +82-2-2123-2825; fax: +82-2-312-2159.

Email address: kwkang75@yonsei.ac.kr (Keonwook Kang)

**<List of Supplementary information>**

**Supplementary discussion 1.** The spatial stress distribution induced by the arrays of misfit dislocations

**Supplementary discussion 2.** The spatial stress distribution induced by the lattice dislocation array

**Supplementary discussion 3.** Effect of lattice dislocation type on the trapping behavior of FCI

**Supplementary discussion 4.** Descriptions of supplementary Movies

1. **The spatial stress distribution induced by the arrays of misfit dislocations [1]**

In order to analyze the influence of the characteristics of misfit dislocation on the lattice dislocation trapping mechanism, the stress field induced by arrays of misfit dislocations was computed using anisotropic linear elasticity solution based on biperiodic Fourier series^1^. To obtain the spatial stress distribution based on anisotropic elasticity theory, it is necessary to define the displacement boundary conditions along the interface in the Fourier series form. So, the wave vector **k** and the position vector **r** for arrays of misfit dislocations were written as

|  | $\text{k}=\text{n}\text{p}_{\text{1}}^{\text{x}}=\text{n}\text{p}_{\text{1}}^{o}/\left\Vert\text{p}_{\text{1}}^{o} \right\Vert^{2}$ and $\text{r}=\text{x}_{\text{1}}\text{p}_{\text{1}}^{\text{o}}$ | (1) |
| --- | --- | --- |

where $\text{p}_{\text{1}}^{\text{o}}$ and $\text{p}_{\text{1}}^{\text{x}}$ are the O-lattice vector and its reciprocal vector, respectively. The $\text{p}_{\text{1}}^{\text{o}}$ and $\text{p}_{\text{1}}^{\text{x}}$ are satisfying the $\text{p}_{\text{1}}^{\text{o}}\cdot\text{p}_{\text{1}}^{\text{x}}=\text{1}$. $\text{x}_{\text{i}}$ is the position in *x_i_*-axis in this study. The detailed geometry of the misfit dislocation and its O-lattice vector can be found in Figure 1-b. To compute the elastic field of anisotropic elastic solid generated by the arrays of straight misfit dislocations, we solved mechanical equilibrium equation as expressed in Equation 2.

|  | $\text{div}\left( \text{C}\text{ }\text{:}\text{ }\text{grad}\text{ }\text{u}_{\text{int}} \right)=\text{0}$ | (2) |
| --- | --- | --- |

where $\text{C}$ is fourth-order the stiffness tensor and $\text{u}_{\text{int}}$ is the displacement field generated by arrays of misfit dislocations. The stiffness tensors for ferrite and cementite phase were given in Table S1 using Voight notation. The general solution satisfying the Equation 2 can be expressed as

|  | $\text{u}_{\text{int}}=\text{u}_{\text{aff}}+\text{u}_{\text{non-aff}}$  $\text{ }\text{ }=\text{u}_{\text{0}}+\text{D}_{\text{c}}\text{ }\text{∙}\text{ }\text{x}+\frac{\text{1}}{\text{i}\text{2}\text{π}}\sum_{\text{k}_{\text{1}}\text{ ≠ 0}} \text{e}^{\text{i}\text{2}\text{π}\text{k}_{\text{1}}\text{x}_{\text{1}}}\text{u}_{\text{k}}\left( \text{x}_{\text{2}} \right)$  where $\text{u}_{\text{k}}\left( \text{x}_{\text{2}} \right)=\text{e}^{\text{i}\text{2}\text{πp}\text{x}_{\text{2}}}\text{a}$ | (3) |
| --- | --- | --- |

where $\text{u}_{\text{aff}}$ and $\text{u}_{\text{non-aff}}$ represent the affine and non-affine displacement fields, respectively. The affine and non-affine displacement fields are given by the coherency strain to satisfy macroscopic geometric compatibility between ferrite and cementite block and the non-uniform displacement to minimize the atomic mismatch between cementite and ferrite atomic layer, respectively. $\text{u}_{\text{0}}$and $\text{D}_{\text{c}}$ are an arbitrary constant displacement vector and elastic distortion field tensor, respectively.$\text{ x}$ is the position vector. *i* is the unit imaginary number defined by *i* = $\sqrt{\text{-1}}$. *k*_1_ is non-zero wave vector component. $\text{p}$ and $\text{a}$ are eigenvalue and its eigenvector, respectively. By combining Equation 2 and 3, we can get the Equation 4 as follows:

|  | $\left( {\text{k}_{\text{1}}^{\text{2}}\text{W}}_{\text{1}}+\text{k}_{\text{1}}\left( \text{W}_{\text{2}}+\text{W}_{\text{2}}^{\text{t}} \right)\text{p}+\text{W}_{\text{3}}\text{p}^{\text{2}} \right)\text{ }\text{∙}\text{ }\text{a}=\text{0}$  where  $\text{W}_{\text{1}}=\left[ \begin{matrix} \text{c}_{\text{11}} & \text{c}_{\text{16}} & \text{c}_{\text{15}} \\ \text{c}_{\text{16}} & \text{c}_{\text{66}} & \text{c}_{\text{56}} \\ \text{c}_{\text{15}} & \text{c}_{\text{56}} & \text{c}_{\text{55}} \end{matrix} \right]$, $\text{W}_{\text{2}}=\left[ \begin{matrix} \text{c}_{\text{16}} & \text{c}_{\text{12}} & \text{c}_{\text{14}} \\ \text{c}_{\text{66}} & \text{c}_{\text{26}} & \text{c}_{\text{46}} \\ \text{c}_{\text{56}} & \text{c}_{\text{25}} & \text{c}_{\text{45}} \end{matrix} \right]$, and $\text{W}_{\text{3}}=\left[ \begin{matrix} \text{c}_{\text{66}} & \text{c}_{\text{26}} & \text{c}_{\text{46}} \\ \text{c}_{\text{26}} & \text{c}_{\text{22}} & \text{c}_{\text{24}} \\ \text{c}_{\text{46}} & \text{c}_{\text{24}} & \text{c}_{\text{44}} \end{matrix} \right]$ | (4) |
| --- | --- | --- |

where $\text{W}_{\text{1}}$, $\text{W}_{\text{2}}$ and $\text{W}_{\text{3}}$ are matrices related to the elastic properties. *c_ij_* represents the component of the stiffness tensor using Voight notation. Superscript ^t^ indicates the matrix transpose. In order to obtain a non-trivial solution, we solved the sextic eigenvalue problem with unknown $\text{p}$ derived from Equation 4. Therefore, we determined the six eigenvalues $\text{p}^{\text{α}}$ and its corresponding eigenvectors $\text{a}^{\text{α}}$. For convenience, we set the first three eigenvalues $\text{p}^{\text{α}}\text{,} \text{α}\text{ = 1 to 3}$ to eigenvalues with a positive imaginary number. The remaining three eigenvalues were rearranged to satisfying the $\text{p}^{\text{α}\text{ }\text{+}\text{ }\text{3}}=\text{p}_{*}^{\text{α}}\text{,}$ for $\text{α}\text{ = 1 to 3}$. The asterisk indicates the complex conjugate operator. In addition, the eigenvectors $\text{a}^{\text{α}}$ were rearranged with respect to its corresponding eigenvalues $\text{p}^{\text{α}}$ and these eigenvectors also satisfy the $\text{a}^{\text{α}\text{ }\text{+}\text{ }\text{3}}=\text{a}_{*}^{\text{α}}\text{,}$ for $\text{α}\text{ = 1 to 3}$. From the computed eigenvalues $\text{p}^{\text{α}}$ and eigenvectors $\text{a}^{\text{α}}$, the complete solution of the displacement field $\text{u}_{\text{int}}$ generated by the arrays of misfit dislocation can be expressed as

|  | $\text{u}_{\text{int}}=\text{u}_{\text{0}}+\text{D}_{\text{c}}\text{ }\text{∙}\text{ }\text{x}+\frac{\text{1}}{\text{i}\text{2}\text{π}}\sum_{\text{k}_{\text{1}}\text{ ≠ 0}} \text{e}^{\text{i}\text{2}\text{π}\text{k}_{\text{1}}\text{x}_{\text{1}}}\sum_{\text{α}\text{ = 1}}^{\text{3}} \text{λ}^{\text{α}}\text{e}^{\text{i}\text{2}\text{π}\text{p}^{\text{α}}\text{x}_{\text{2}}}\boldsymbol{a}^{\text{α}}+\text{ξ}^{\text{α}}\text{e}^{\text{i}\text{2}\text{π}\text{p}_{\text{*}}^{\text{α}}\text{x}_{\text{2}}}\boldsymbol{a}_{*}^{\text{α}}$ | (5) |
| --- | --- | --- |

where $\text{λ}^{\text{α}}$ and $\text{ξ}^{\text{α}}$ represent unknown coefficients which can be determined by the displacement and traction boundary conditions. In order to compute the stress field induced by arrays of misfit dislocations, we assume the arrays of misfit dislocations as periodically implemented multiple Volterra dislocations along the FCI. The given problem should satisfy the two displacement boundary conditions. One is a convergence of elastic fields, and the other is that the top and bottom atomic layers at the interface experience the stepwise displacement jump. According to Saint Venant’s principle, the non-affine displacement field $\text{u}_{\text{non-aff}}$ should be converged when *x*_2_ goes to positive/negative infinity ($\text{x}_{\text{2}}\text{ }\text{→}\text{ }\text{±∞}$). So, it determines the unknown coefficients as ${}_{\text{C}}{\text{ξ}^{\text{α}}}\text{ }\text{=}\text{ }\text{0}$ and ${}_{\text{F}}{\text{λ}^{\text{α}}}\text{ }\text{=}\text{ }\text{0}$. In addition, the arrays of Volterra dislocations at the interface give rise to stepwise displacement jump. So, the disregistry of Volterra dislocations can be represented using step functions as depicted in Figure 2-a. The disregistry represents the relative displacement between the bottom surface of cementite and top surface of ferrite. Each step height and interval of step function represent the magnitude of Burgers vector *b*_int_ = $\left\| \text{b}_{\text{int}} \right\|$ and period of dislocation *d*_int_. So, the disregistry at the interface (*x*_2_ = 0) is expressed as

|  | $\text{∆}\text{u}_{\text{int}}\left( \text{x}_{\text{1}}\text{,}\text{ }\text{x}_{\text{3}} \right)={}_{\text{C}}{\text{u}_{\text{int}}}\left( \text{x}_{\text{1}}\text{,}\text{ }\text{x}_{\text{3}} \right)-{}_{\text{F}}{\text{u}_{\text{int}}}\left( \text{x}_{\text{1}}\text{,}\text{ }\text{x}_{\text{3}} \right)$  $\text{ } =\text{∆}\text{u}_{\text{0}}+\left. \left( {}_{\text{C}}{\text{D}_{\text{c}}}-{}_{\text{F}}{\text{D}_{\text{c}}} \right)\text{ }\text{∙}\text{ }\text{x} \right\vert_{\text{x}_{\text{2}}=\text{0}} +\frac{\text{1}}{\text{i}\text{2}\text{π}}\sum_{\text{k}_{\text{1}}\text{ ≠ 0}} \text{e}^{\text{i}\text{2}\text{π}\text{k}_{\text{1}}\text{x}_{\text{1}}}\sum_{\text{α}\text{ = 1}}^{\text{3}} {}_{\text{C}}{\text{λ}^{\text{α}}}{}_{\text{C}}{\boldsymbol{a}^{\text{α}}}-{}_{\text{F}}{\text{ξ}^{\text{α}}}{}_{\text{F}}{\boldsymbol{a}_{*}^{\text{α}}}$ | (6) |
| --- | --- | --- |

where $\text{∆}\text{u}_{\text{int}}$represents the disregistry at the FCI. ${}_{\text{C}}{\text{u}_{\text{int}}}$ and ${}_{\text{F}}{\text{u}_{\text{int}}}$ are a displacement of cementite and ferrite at the interface, respectively. Moreover, the disregistry $\text{∆}\text{u}_{\text{int}}\left( \text{x}_{\text{1}}\text{,}\text{ }\text{x}_{\text{3}} \right)$ can be decomposed into affine $\text{∆}\text{u}_{\text{aff}}$ and non-affine $\text{∆}\text{u}_{\text{non-aff}}$ terms. Each disregistry is expressed as

|  | $\text{∆}\text{u}_{\text{aff}}\left( \text{x}_{\text{1}}\text{,}\text{ }\text{x}_{\text{3}} \right)\text{= }\text{∆}\text{u}_{\text{0}}+\left. \left( {}_{\text{C}}{\text{D}_{\text{c}}}-{}_{\text{F}}{\text{D}_{\text{c}}} \right)\text{ }\text{∙}\text{ }\text{x} \right\vert_{\text{x}_{\text{2}}=\text{0}}$ | (7) |
| --- | --- | --- |
|  | $\text{∆}\text{u}_{\text{non-aff}}\left( \text{x}_{\text{1}}\text{,}\text{ }\text{x}_{\text{3}} \right)=\frac{\text{1}}{\text{i}\text{2}\text{π}}\sum_{\text{k}_{\text{1}}\text{ ≠ 0}} \text{e}^{\text{i}\text{2}\text{π}\text{k}_{\text{1}}\text{x}_{\text{1}}}\sum_{\text{α}\text{ = 1}}^{\text{3}} {}_{\text{C}}{\text{λ}^{\text{α}}}{}_{\text{C}}{\boldsymbol{a}^{\text{α}}}-{}_{\text{F}}{\text{ξ}^{\text{α}}}{}_{\text{F}}{\boldsymbol{a}_{*}^{\text{α}}}$ | (8) |

where $\text{∆}\text{u}_{\text{0}}=-\text{b}_{\text{int}}\text{/2}$ is chosen, without loss of generality. $\text{b}_{\text{int}}$ is the Burgers vector of misfit dislocation. The affine disregistry $\text{∆}\text{u}_{\text{aff}}$ represents the uniform macroscopic distortion by distribution of misfit dislocations with infinitesimal Burgers vector and line spacing. The non-affine disregistry $\text{∆}\text{u}_{\text{non-}\text{aff}}$ represents the relative displacement between the interface atoms to form the arrays of Volterra dislocations during relaxation process. The relative displacement by the arrays of Volterra dislocation at the interface is expressed as

|  | $\text{∆}\text{u}_{\text{non-aff}}\left( \text{x}_{\text{1}}\text{,}\text{ }\text{x}_{\text{3}} \right)=\sum_{\text{n}\text{ = 1}}^{\text{∞}} -\frac{\text{b}_{\text{int}}}{\text{nπ}}\sin\text{2}\text{π}\text{k}_{\text{1}}\text{x}_{\text{1}}$ | (9) |
| --- | --- | --- |

Table S1. Elastic modulus for ferrite and cementite at 300 K (unit: GPa).

|  | *C*_11_ | *C*_22_ | *C*_33_ | *C*_12_ | *C*_23_ | *C*_13_ | *C*_44_ | *C*_55_ | *C*_66_ |
| --- | --- | --- | --- | --- | --- | --- | --- | --- | --- |
| Fe_3_C | 248 | 309 | 288 | 120 | 158 | 146 | 93 | 79 | 33 |
| α-Fe | 222 | | | 145 | | | 127 | | |

The non-affine disregistry gives sawtooth function by Fourier sine series analysis. Thus, the Equation 8 and 9 lead the linear equations with complex coefficients and variables. The linear equation is expressed as

|  | $\text{Re}\sum_{\text{α}\text{ = 1}}^{\text{3}} {}_{\text{C}}{\text{λ}^{\text{α}}}{}_{\text{C}}{\boldsymbol{a}^{\text{α}}}-{}_{\text{F}}{\text{ξ}^{\text{α}}}{}_{\text{F}}{\boldsymbol{a}_{*}^{\text{α}}}=-\text{2}\text{b}_{\text{int}}\text{/}\text{n}\text{, }\text{ }\text{for }\text{n}\text{ }\text{≥}\text{ }\text{1}$  $\text{Im}\sum_{\text{α}\text{ = 1}}^{\text{3}} {}_{\text{C}}{\text{λ}^{\text{α}}}{}_{\text{C}}{\mathbf{a}^{\text{α}}}-{}_{\text{F}}{\text{ξ}^{\text{α}}}{}_{\text{F}}{\boldsymbol{a}_{*}^{\text{α}}}=\text{0}\text{, }\text{ }\text{for }\text{n}\text{ }\text{≥}\text{ }\text{1}$ | (10) |
| --- | --- | --- |

where Re and Im represent the real and imaginary part of a complex number. The FCI should satisfy the mechanical equilibrium condition along the interface. So, the net traction along the interface should be zero. The traction boundary condition at the interface ($\text{x}_{\text{2}}=\text{0}$) is expressed as

|  | ${}_{\text{C}}{\text{σ}_{\text{int}}}\left( \text{x}_{\text{1}}\text{,}\text{ }\text{x}_{\text{3}} \right)\text{ }\text{∙}\text{ }\text{n}\mathbf{=}{}_{\text{F}}{\text{σ}_{\text{int}}\left( \text{x}_{\text{1}}\text{,}\text{ }\text{x}_{\text{3}} \right)}\text{ }\text{∙}\text{ }\text{n}$ | (11) |
| --- | --- | --- |

where $\text{σ}_{\text{int}}$ represents a short-range stress field induced by arrays of misfit dislocations. **n** is the interface normal. For each phase, we can compute traction along the interface using the interface normal **n**, non-affine displacement field $\text{u}_{\text{non-aff}}$ and fourth order stiffness tensor **C**. The traction along the interface is expressed as

|  | $\text{σ}_{\text{int}}\left( \text{x}_{\text{1}}\text{, }\text{x}_{\text{3}} \right)\text{ ∙ }\text{n}\mathbf{=}\text{sign(}\text{x}_{\text{2}}\text{)}\sum_{\text{k}_{\text{1}}\text{ ≠ 0}} \text{e}^{\text{i}\text{2}\text{π}\text{k}_{\text{1}}\text{x}_{\text{1}}}\sum_{\text{α}\text{ = 1}}^{\text{3}} \text{λ}^{\text{α}}\text{h}^{\text{α}}+\text{ξ}^{\text{α}}\text{h}_{*}^{\text{α}}$ | (12) |
| --- | --- | --- |

where $\text{sign}$ represents the sign function. $\text{h}^{\text{α}}$ are complex vectors related to elastic modulus *c_ij_*, wave vector component *k*_1_, eigenvalues $\text{p}^{\text{α}}$ and the eigenvectors $\boldsymbol{a}^{\text{α}}$. $\text{h}^{\text{α}}$ is computed by

|  | $\text{h}^{\text{α}}= \left( \text{k}\text{1}\text{W}_{\text{2}}^{\text{t}}+\text{p}^{\text{α}}\text{W}_{\text{3}} \right)\text{ ∙ }\boldsymbol{a}^{\text{α}}=-\frac{\text{1}}{\text{p}^{\text{α}}}\left( \text{k}_{\text{1}}^{2}\text{W}_{\text{1}}+\text{k}\text{1}\text{p}^{\text{α}}\text{W}_{\text{2}} \right)\text{ ∙ }\boldsymbol{a}^{\text{α}}$ | (13) |
| --- | --- | --- |

Thus, the Equation 11 through 13 also lead the linear equations with complex coefficients and variables. The linear equation is expressed as

|  | $\text{Re}\sum_{\text{α}\text{ = 1}}^{\text{3}} {}_{\text{C}}{\text{λ}^{\text{α}}}{}_{\text{C}}{\text{h}^{\text{α}}}-{}_{\text{F}}{\text{ξ}^{\text{α}}}{}_{\text{F}}{\text{h}_{*}^{\text{α}}}=\text{0}$  $\text{Im}\sum_{\text{α}\text{ = 1}}^{\text{3}} {}_{\text{C}}{\text{λ}^{\text{α}}}{}_{\text{C}}{\text{h}^{\text{α}}}-{}_{\text{F}}{\text{ξ}^{\text{α}}}{}_{\text{F}}{\text{h}_{*}^{\text{α}}}=\text{0}$ | (14) |
| --- | --- | --- |

From the displacement and traction boundary condition, the unknown complex coefficients ${}_{\text{C}}{\text{λ}^{\text{α}}}$ and ${}_{\text{F}}{\text{ξ}^{\text{α}}}$ can be obtained by solving the both Equation 10 and 14. Therefore, we obtain the spatial stress distribution in ferrite ${}_{\text{F}}{\text{σ}_{\text{int}}}\left( \text{x}_{\text{1}}\text{,}\text{ }\text{x}_{\text{2}}\text{,}\text{ }\text{x}_{\text{3}} \right)$ and cementite layer ${}_{\text{C}}{\text{σ}_{\text{int}}}\left( \text{x}_{\text{1}}\text{,}\text{ }\text{x}_{\text{2}}\text{,}\text{ }\text{x}_{\text{3}} \right)$ by ${}_{\text{F}}{\text{σ}_{\text{int}}}\left( \text{x}_{\text{1}}\text{,}\text{ }\text{x}_{\text{2}}\text{,}\text{ }\text{x}_{\text{3}} \right)=\text{C}\text{ }\text{:}\text{ }\text{grad}\text{ }{}_{\text{F}}{\text{u}_{\text{int}}}\left( \text{x}_{\text{1}}\text{,}\text{ }\text{x}_{\text{2}}\text{,}\text{ }\text{x}_{\text{3}} \right)$ and ${}_{\text{C}}{\text{σ}_{\text{int}}}\left( \text{x}_{\text{1}}\text{,}\text{ }\text{x}_{\text{2}}\text{,}\text{ }\text{x}_{\text{3}} \right)=\text{C}\text{ }\text{:}\text{ }\text{grad}\text{ }{}_{\text{C}}{\text{u}_{\text{int}}}\left( \text{x}_{\text{1}}\text{,}\text{ }\text{x}_{\text{2}}\text{,}\text{ }\text{x}_{\text{3}} \right)$, respectively.

1. **The spatial stress distribution induced by the lattice dislocation array [2]**

The spatial stress distribution generated by a straight lattice dislocation within the ferrite/cementite bilayer can be obtained by solving anisotropic linear elasticity problem due to a straight single Volterra dislocation in infinite bicrystals^2^. However, introducing a single lattice dislocation in MD simulation (Figure 1-a) can be interpreted as an array of dislocations along *x*-direction with respect to periodic boundary condition (PBC) of the simulation box. So, in order to compare anisotropic linear elasticity solution with MD simulation results, we solved anisotropic linear elasticity problem considering an array of straight single Volterra dislocations with period length (L) equal to MD simulation box length along *x*-direction in infinite bicrystals^2^. In this study, the line orientation **ξ**_lat_ of the lattice dislocation was set parallel to the direction vector **t** (**ξ**_lat_ || **t**), and we defined direction vector **m** and **n** as vectors perpendicular to the direction vector **t**. For easy analysis, we set the **m**, **n**, and **t** vectors as vectors parallel to the *x*_1_, *x*_2_ and *x*_3_-axis, respectively. If the lattice dislocation array is implemented in the ferrite layer, the displacement field in the ferrite layer ${}_{\text{F}}{\text{u}_{\text{lat}}}$ and in cementite layer ${}_{\text{C}}{\text{u}_{\text{lat}}}$ is expressed as

|  | ${}_{\text{F}}{\text{u}_{\text{lat}}}={}_{\text{F}}{\text{u}_{\text{inf}}}\boldsymbol{+}{}_{\text{F}}{\text{u}_{\text{img}}}$  ${}_{\text{C}}{\text{u}_{\text{lat}}}={}_{\text{C}}{\text{u}_{\text{img}}}$ | (15) |
| --- | --- | --- |

where ${}_{\text{F}}{\text{u}_{\text{inf}}}$ represents the displacement field solution of the infinite ferrite crystal. ${}_{\text{F}}{\text{u}_{\text{img}}}$ and ${}_{\text{C}}{\text{u}_{\text{img}}}$ are the displacement field solution for ferrite and cementite phase from the displacement continuity across the interface and traction free boundary conditions along the interface. So, each displacement field solution is expressed as^2^:

|  | ${}_{\text{F}}{\text{u}_{\text{i}\text{nf}}}=\frac{\text{1}}{\text{L}}Im\left\{ \boldsymbol{A}^{F}\left\langle\cot\left( \pi\frac{\boldsymbol{T}_{*}^{F}-\boldsymbol{S}_{*}^{F}(0)}{L} \right)\left( \delta_{i1}+\boldsymbol{p}_{*}^{F}\delta_{i2} \right) \right\rangle\boldsymbol{q}^{\infty,F} \right\}$  ${}_{\text{F}}{\text{u}_{\text{i}\text{mg}}}=\frac{\text{1}}{\text{L}}Im\sum_{j=1}^{3} \left\{ \boldsymbol{A}^{F}\left\langle\cot\left( \pi\frac{\boldsymbol{T}_{*}^{F}-{\bar{\boldsymbol{S}}}_{j}^{F}(0)}{L} \right)\left( \delta_{i1}+\boldsymbol{p}_{*}^{F}\delta_{i2} \right) \right\rangle\boldsymbol{q}_{j}^{F} \right\}$  ${}_{\text{C}}{\text{u}_{\text{img}}}=\frac{\text{1}}{\text{L}}Im\sum_{j=1}^{3} \left\{ \boldsymbol{A}^{C}\left\langle\cot\left( \pi\frac{\boldsymbol{T}_{*}^{C}-\boldsymbol{S}_{j}^{F}(0)}{L} \right)\left( \delta_{i1}+\boldsymbol{p}_{*}^{C}\delta_{i2} \right) \right\rangle\boldsymbol{q}_{j}^{C} \right\}$  With  $\boldsymbol{T}_{j}^{\text{κ}}=x+p_{j}^{\text{κ}}y$, $\boldsymbol{S}_{j}^{\text{F}}(n)={(X}_{0}-nL)+\boldsymbol{p}_{j}^{\text{F}}Y$ and $\boldsymbol{q}^{\infty,F}={(B^{F})}^{t}\boldsymbol{b}$ | (16) |
| --- | --- | --- |

$\boldsymbol{T}_{j}^{\text{κ}}$ and $\boldsymbol{S}_{j}^{\text{κ}}\left( n \right)$ are complex variables associated with the field and lattice dislocation source points, which the (*x,y*) and (*X_0_,Y*) are arbitrary point and reference lattice dislocation position within the ferrite layer, respectively. The L and ***b*** are the period length of lattice dislocation array and corresponded Burgers vector, respectively. The $\boldsymbol{p}_{j}^{\text{κ}}$ and $\boldsymbol{A}^{\text{κ}}$ are eigenvalues and their corresponding eigenmatrix, respectively, determined by Equation 2. The κ represents the phase of a given position vector $\text{x}$ and can be an F (ferrite) or C (cementite). In Equation (16) an overbar means complex conjugate and t denotes matrix transpose. The $\left\langle\right\rangle$ represent the diagonal matric over *:1,2,3. The $B^{F}$and $A^{F}$ are normalized to satisfying the orthogonality condition expressed as $\boldsymbol{A}^{\text{κ}}.{(\boldsymbol{B}^{\text{κ}})}^{t}+\boldsymbol{B}^{\text{κ}}.{(\boldsymbol{A}^{\text{κ}})}^{t}=\boldsymbol{I}$ where $\text{I}$ is 3 by 3 identity matrix. The$\boldsymbol{q}_{j}^{F}$ and $\boldsymbol{q}_{j}^{C}$ are unknown vectors that can be determined by the continuity conditions for the perfect interface. From the continuity conditions, $\boldsymbol{q}_{j}^{F}$ and $\boldsymbol{q}_{j}^{C}$ are expressed as:

|  | $\boldsymbol{q}_{j}^{\text{F}}=\left( \boldsymbol{A}^{\text{F}} \right)^{-1}\left( \boldsymbol{M}^{\text{F}}+{\bar{\boldsymbol{M}}}^{\text{C}} \right)^{-1}({\bar{\boldsymbol{M}}}^{\text{C}}-{\bar{\boldsymbol{M}}}^{\text{F}}){\bar{\boldsymbol{A}}}^{\text{F}}\boldsymbol{I}_{j}{\bar{\boldsymbol{q}}}^{\infty,F}$  $\boldsymbol{q}_{j}^{\text{C}}=\left( \boldsymbol{A}^{\text{C}} \right)^{-1}\left( {\bar{\boldsymbol{M}}}^{\text{F}}+\boldsymbol{M}^{\text{C}} \right)^{-1}(\boldsymbol{M}^{\text{F}}+{\bar{\boldsymbol{M}}}^{\text{F}})\boldsymbol{A}^{\text{F}}\boldsymbol{I}_{j}\boldsymbol{q}^{\infty,F}$  With  $\boldsymbol{M}^{\text{κ}}=-i\boldsymbol{B}^{\text{κ}}\left( \boldsymbol{A}^{\text{κ}} \right)^{-1}$  $\boldsymbol{I}_{1}=<1,0,0>$; $\boldsymbol{I}_{2}=<0,1,0>$; $\boldsymbol{I}_{3}=<0,0,1>$ | (17) |
| --- | --- | --- |

From Equation 17, the unknown vectors $\boldsymbol{q}_{j}^{\text{F}}$ and $\boldsymbol{q}_{j}^{\text{F}}$ are determined. From the displacement field solution, we obtain both the infinite stress field solution ${}_{\text{κ}}{\text{σ}_{\text{inf}}}\left( \text{x}_{\text{1}}\text{,}\text{ }\text{x}_{\text{2}}\text{,}\text{ }\text{x}_{\text{3}} \right)$ and image stress field solution ${}_{\text{κ}}{\text{σ}_{\text{img}}}\left( \text{x}_{\text{1}}\text{,}\text{ }\text{x}_{\text{2}}\text{,}\text{ }\text{x}_{\text{3}} \right)$ by using:

|  | ${}_{\text{κ}}{\text{σ}_{\text{inf}}}\left( \text{x}_{\text{1}}\text{,}\text{ }\text{x}_{\text{2}}\text{,}\text{ }\text{x}_{\text{3}} \right)={}_{\text{κ}}\text{C}\text{ }\text{:}\text{ }\text{grad}\text{ }{}_{\text{κ}}{\text{u}_{\text{inf}}}\left( \text{x}_{\text{1}}\text{,}\text{ }\text{x}_{\text{2}}\text{,}\text{ }\text{x}_{\text{3}} \right)$  ${}_{\text{κ}}{\text{σ}_{\text{img}}}\left( \text{x}_{\text{1}}\text{,}\text{ }\text{x}_{\text{2}}\text{,}\text{ }\text{x}_{\text{3}} \right)={}_{\text{κ}}\text{C}\text{ }\text{:}\text{ }\text{grad}\text{ }{}_{\text{κ}}{\text{u}_{\text{img}}}\left( \text{x}_{\text{1}}\text{,}\text{ }\text{x}_{\text{2}}\text{,}\text{ }\text{x}_{\text{3}} \right)$ | (18) |
| --- | --- | --- |

Therefore, we compute the stress field solution in ferrite ${}_{\text{F}}{\text{σ}_{\text{lat}}}\left( \text{x}_{\text{1}}\text{,}\text{ }\text{x}_{\text{2}}\text{,}\text{ }\text{x}_{\text{3}} \right)$ and cementite ${}_{\text{C}}{\text{σ}_{\text{lat}}}\left( \text{x}_{\text{1}}\text{,}\text{ }\text{x}_{\text{2}}\text{,}\text{ }\text{x}_{\text{3}} \right)$ layers by using:

|  | ${}_{F}{\boldsymbol{\sigma}_{\mathrm{lat}}}\left( x_{1}, x_{2}, x_{3} \right)={}_{F}{\boldsymbol{\sigma}_{\inf}}\left( x_{1}, x_{2}, x_{3} \right)+{}_{F}{\boldsymbol{\sigma}_{\mathrm{img}}}\left( x_{1}, x_{2}, x_{3} \right)$  ${}_{\text{C}}{\text{σ}_{\text{lat}}}\left( \text{x}_{\text{1}}\text{,}\text{ }\text{x}_{\text{2}}\text{,}\text{ }\text{x}_{\text{3}} \right)={}_{\text{C}}{\text{σ}_{\text{img}}}\left( \text{x}_{\text{1}}\text{,}\text{ }\text{x}_{\text{2}}\text{,}\text{ }\text{x}_{\text{3}} \right)$ | (19) |
| --- | --- | --- |

1. **Effect of lattice dislocation type on the trapping behavior of FCI**

To compare the effect of Burgers vector type on the trapping behavior of FCI, we split the b_lat_ = [0.84 -1.16 2.01] Å into the b_edge_ = [0.84 -1.16 0] Å and b_screw_ = [0 0 2.01] Å with respect to the dislocation line orientation **ξ**_lat_ = [0.00 0.00 1.00] and recalculate the P-K forces:


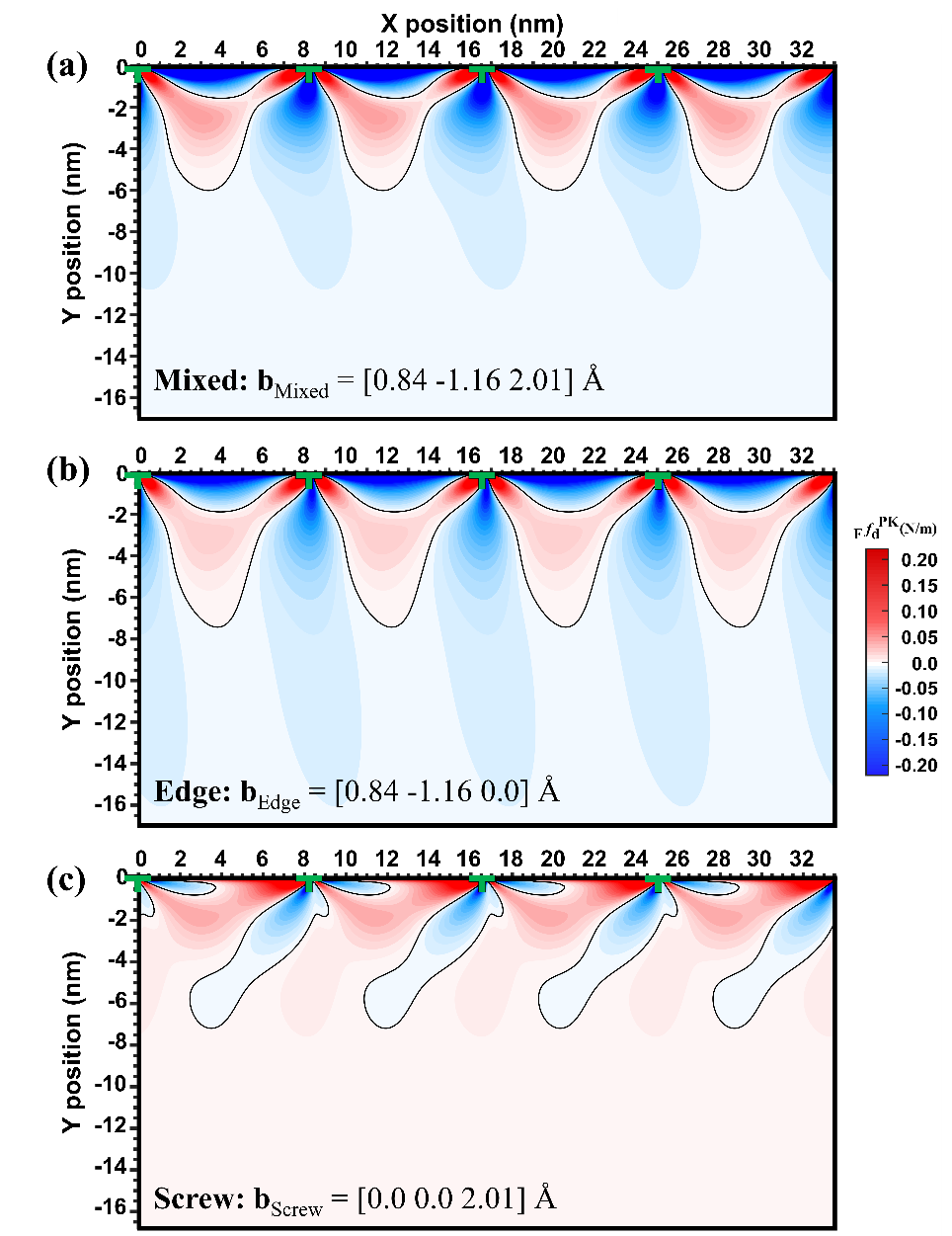


Figure S1. Contour plot of resolved P-K force within the ferrite layer with (a) a mixed lattice dislocation of b_lat_ = [0.84 -1.16 2.01] Å (replotting of Fig. 3a), (b) an edge lattice dislocation with b_edge_ = [0.84 -1.16 0] Å and (c) a screw lattice dislocation with b_screw_ = [0 0 2.01] Å.

Comparison of P-K forces in Fig. S1 reveals a predominant effect of edge component on the trapping ability of FCI due to its direction parallel with the direction vector in the slip plane (**d**).

1. **Descriptions of supplementary Movies.**

The five supplementary movies are labeled according to their initial position of lattice dislocation (*x^*^, y^*^*) as shown in Figure S2. The *x^*^* and *y*^*^ represent the initial location of lattice dislocation along *x*- and *y*- directions with respect to the target box with 18$\times$11 grid points starting from 0. Among all initial positions, only the positions marked by the diamond symbol show oscillating around the ${}_{\text{F}}{\text{f}_{\text{d}}^{\text{PK}}}=0$ borderline during MD simulation up to 600 ps.


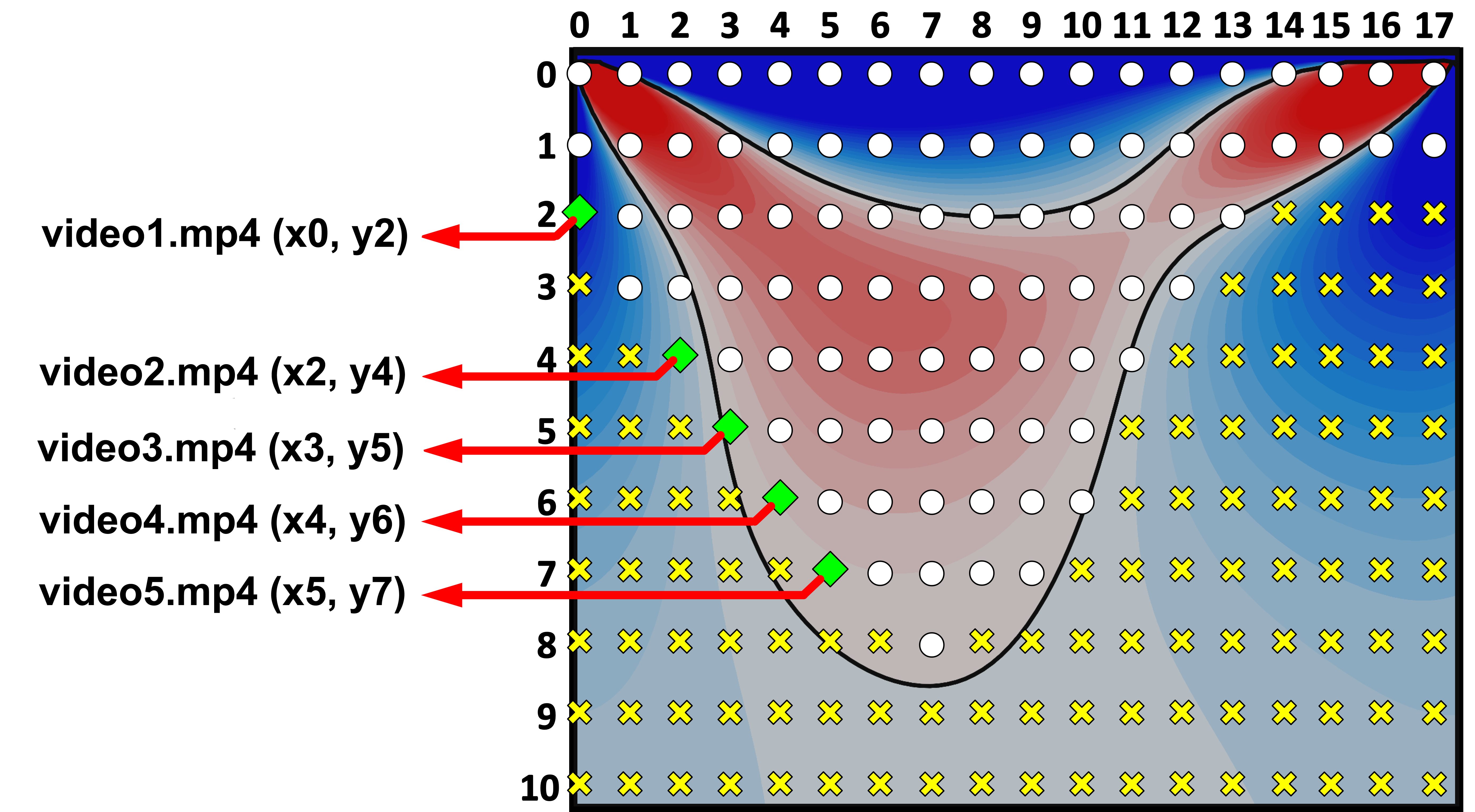


Figure S2. The initial position of lattice dislocation in the supplementary movies.

**References**

1 Vattré, A. J. & Demkowicz, M. J. Determining the Burgers vectors and elastic strain energies of interface dislocation arrays using anisotropic elasticity theory. *Acta Materialia* **61**, 5172-5187, doi:10.1016/j.actamat.2013.05.006 (2013).

2 Chu, H. & Pan, E. Elastic fields due to dislocation arrays in anisotropic bimaterials. *International Journal of Solids and Structures* **51**, 1954-1961, doi:10.1016/j.ijsolstr.2014.02.001 (2014).
